# Developmental changes in reading do not alter the development of visual processing skills: an application of explanatory item response models in grades K-2

**DOI:** 10.3389/fpsyg.2015.00116

**Published:** 2015-02-11

**Authors:** Kristi L. Santi, Paulina A. Kulesz, Shiva Khalaf, David J. Francis

**Affiliations:** ^1^University of HoustonHouston, TX, USA; ^2^College of Education, University of HoustonHouston, TX, USA; ^3^Department of Psychology, University of HoustonHouston, TX, USA

**Keywords:** visual motor integration, visual processing, reading development, language based reading predictors, early reading skills

## Abstract

Visual processing has been widely studied in regard to its impact on a students’ ability to read. A less researched area is the role of reading in the development of visual processing skills. A cohort-sequential, accelerated-longitudinal design was utilized with 932 kindergarten, first, and second grade students to examine the impact of reading acquisition on the processing of various types of visual discrimination and visual motor test items. Students were assessed four times per year on a variety of reading measures and reading precursors and two popular measures of visual processing over a 3-year period. Explanatory item response models were used to examine the roles of person and item characteristics on changes in visual processing abilities and changes in item difficulties over time. Results showed different developmental patterns for five types of visual processing test items, but most importantly failed to show consistent effects of learning to read on changes in item difficulty. Thus, the present study failed to find support for the hypothesis that learning to read alters performance on measures of visual processing. Rather, visual processing and reading ability improved together over time with no evidence to suggest cross-domain influences from reading to visual processing. Results are discussed in the context of developmental theories of visual processing and brain-based research on the role of visual skills in learning to read.

## INTRODUCTION

Reading, an everyday task that is essential to success, is a complex developmental activity. Reading is interwoven with other developmental tasks such as attention, memory, and language. Researchers who focus on the cognitive aspects of learning to read have posited numerous theoretical models to describe the process. The simple view of reading (SVR: Gough and Tunmer, 1986) is one popular theoretical framework that stipulates that reading consists of two components: decoding and linguistic comprehension. The model is silent about the complex processes that enable decoding and linguistic comprehension, which together have been the focus of much reading research over the past 30 years. It is generally accepted that the decoding aspect of the model is itself developmental, building from foundational skills in the phonological code to more advanced reading skills that incorporate orthographic processes and automaticity in execution of decoding routines that together allow the reader to rapidly access word-level information encoded in print. The linguistic comprehension aspect of the model encompasses the reader’s ability to rapidly retrieve the meanings of words and deduce both sentence- and discourse-level interpretations. The Construction-Integration model of van Dijk and Kintsch (1983) and the Landscape Model of van den Broek et al. (2005) are two of the most widely cited cognitive models for explaining how readers make sense of text, i.e., for elaborating the cognitive and linguistic processes involved in the linguistic comprehension component of the SVR. However, these models largely describe the process of skilled reading and are not generally recognized as developmental models of reading. That is, they do not attempt to capture the quantitative and qualitative changes that characterize reading as individuals develop from non-readers, to individuals learning to read, and ultimately to individuals reading to learn.

What we know about how children learn to read is well documented. Children must learn the alphabetic principal in order to become proficient readers (see Adams, 1994; Snow et al., 1998; National Institute of Child Health, and Human Development [NICHD], 2000 for a comprehensive review of the syntheses of the research). The skills consistently found essential for students to learn are often categorized into five main areas: phonemic awareness, phonics, vocabulary, fluency, and comprehension (National Institute of Child Health, and Human Development [NICHD], 2000). Several reports and books have compiled the research into easily accessible readings for educators, parents, and researchers. A review of three separate meta-analyses (Hammill, 2004) was conducted to determine the abilities most highly related to reading achievement. This review found that the three prior meta-analyses were consistent with the research reviewed by the National Research Council committee on early reading problems, which was headed by Snow, Burns, and Griffin, and the National Institute of Health’s National Reading Panel. These reports are noteworthy for many reasons, but especially in the context of the present study for what they conclude about the relatively minor role played by visual processes in learning to read.

Peer reviewed research on the developmental trends relating reading ability to changes in visual processing as measured by tests of visual motor integration can be traced back to the 1960s. Much of this research has been correlational in nature and has found limited evidence of a role for visual processing in explaining individual differences in reading acquisition (Birch and Belmont, 1965; Beery, 1967; Busch, 1980; Wright and DeMers, 1982; Margolese and Kline, 1999). On balance these studies have reached similar conclusions which point to a limited role for visual motor skills in reading achievement, and a much stronger role for language based measures such as letter names and sounds, vocabulary, phonological skills, and language comprehension.

More recent research has found evidence that learning to read might alter individuals’ processing of visual information. Research coming out of numerous laboratories engaged in functional magnetic resonance imaging (fMRI) has provided compelling evidence that the acquisition of reading may alter specific brain areas involved in the processing of visual information, including words and faces. For example, Olulade et al. (2013) investigated relationships between brain activity in area V5/MT during visual motion processing and reading ability by providing a group of dyslexic children with a phonologically based reading intervention. Using within-person controls, Olulade et al. (2013) found that exposing dyslexic children to the reading intervention resulted in better reading performance and greater activity in area V5/MT during visual motion perception. The authors concluded that reading acquisition has a positive influence on visual development, as demonstrated by the increase in right V5/MT activity after reading gains in children with dyslexia. In another similar study, using fMRI, Dehaene et al. (2010), measured the effect of reading performance on visual responses in the visual word form area (VWFA) – a specific brain site in left occipito-temporal cortex, which has been identified in numerous studies using fMRI and magneto encephalography to change following reading intervention in poor readers (see Pugh et al., 2000; Papanicolaou et al., 2003). Dehaene et al. (2010) reported that literacy enhanced left fusiform activation, and also broadly enhanced visual responses in fusiform and occipital cortex, extending to area V1. Simply put, these findings suggest that learning to read strengthens cortical networks for vision and language. Furthermore, the findings replicated other studies using brain neuroimaging in normal and dyslexic children to show that, with reading acquisition, the VWFA, starts to respond to orthographic stimuli in the learned script (Shaywitz et al., 2002; Maurer et al., 2006).

While these studies provide valuable insight into the relationship between reading and vision, there are several important features to these studies that must be kept in mind in considering whether learning to read affects visual processing skills. First, many studies that have examined brain related changes to learning to read have either compared dyslexic individuals to typical readers, have studied changes in dyslexic individuals following reading intervention, or have compared readers and non-readers. That is, none of these studies have examined, longitudinally, changes in the brains of typically developing individuals as they have learned to read over an extended developmental period. While it is compelling to generalize the changes seen in the brains of dyslexic children as they learn to read to changes in the brains of typically developing children as they learn to read, doing so requires that we ignore, or at least treat as immaterial, the differences between children with and without dyslexia that exist prior to the onset of reading intervention. Additionally, even if one accepts that the changes/differences observed in these studies generalize to typically developing individuals as they learn to read, the question remains whether these effects seen via brain imaging techniques have consequences at more macro levels of behavior. That is, do these changes that result from learning to read impact how individuals process visual information on educational and neuropsychological tests.

The current study attempts to answer this latter question. That is, the current study explores the impact of the development of early reading skills on the visual processing skills of children as measured on standard educational and neuropsychological tests of visual discrimination and visual-motor processing. To examine this question, we must take into account that both reading and visual processing skills evolve as children mature. The development of reading progresses from early manipulation of the sound structure of language to acquisition of the alphabetic principle (i.e., the bi-directional mapping of sound to print and print to sound), to the development of accurate and fluent decoding and comprehension. Likewise, visual discrimination and visual motor skills are not static, but develop throughout childhood.

### THE SEQUENCE OF DEVELOPMENT OF VISUAL MOTOR SKILLS

Children acquire the ability to copy figures in a predictable order from circles to squares to triangles and diamonds at ages three, four, five, and seven, respectively (Rand, 1973). Other features of visual stimuli to which children develop sensitivity as their visual skills develop concern the orientation of stimuli, their visual complexity (i.e., their richness in detail), and their angularity (Beery, 1968a,b). These features have received varying degrees of attention in research on the development of visual, and visual-motor skills. For example, stimuli are known to be more difficult to process for children when they are presented at an oblique orientation, rather than vertically or horizontally (Gibson et al., 1962; Beery, 1968a; Appelle, 1972). Similarly, increasing the complexity of visual stimuli (i.e., increasing the number of sides and angles) increases the difficulty that children have in recognizing, reproducing, or matching them. Angularity also affects the difficulty of visual stimuli, with more acute angles creating greater difficulty for children (Graham et al., 1960), although Beery (1968a) has found that acute angles (especially 45°angles) are overestimated, whereas obtuse angles (especially 135°angles) are underestimated (Piaget, 1949). Moreover, Beery (1968a), has shown that these features interact in their effects on children’s ability to process visual information.

Researchers have also used advanced psychometric modeling techniques, such as factor analysis, to investigate the development of visual motor skills (Polubinski et al., 1986; Brown et al., 2009). Unfortunately, neither of these studies examined differences in children’s performance or differences in the factor structure of tests as children transitioned from being non-readers to readers, or from being beginning readers to skilled readers. If the development of reading affects the processing of visual information, it stands to reason that children’s status as readers might affect how they approach items on a test of visual discrimination or visual-motor integration (VMI) such as the recognition–discrimination test or the Beery VMI. Whether this change in processing would manifest itself as differences in the factor structure/dimensionality of the test or as shifts in the difficulty of test items is not clear. Certainly, changes in the factor structure/dimensionality of a test as a function of changes in students status as readers cannot be explained as simple shifts in the ability distribution of the latent ability measured by the test of visual discrimination or visual-motor skill, whereas changes in item difficulties suggest that performance on test items is changing as a function of the change in status, but not necessarily the nature of the thing being measured.

The present study evaluated the role of reading in the development of visual processing skills using a large longitudinal data set and advances in psychometric/statistical modeling known as explanatory item response models (de Boeck and Wilson, 2004) to examine changes in visual processing associated with learning to read. Using explanatory item response models, discussed below, this study expects to show that phonological skills and the development of phonological awareness (PA), which anticipate the onset of reading acquisition, do not influence performance on test items measuring visual processing skill, either directly, or through interaction with item features that serve to explain item difficulties for visual processing items. It is also expected that measures of rapid naming, decoding, decoding fluency, and spelling, which is closely tied to the development of automated decoding skills, will be most influential in explaining item difficulties of visual processing items, and to predict changes in item difficulties over time, as well as to explain changes in the effects of item features on item difficulty that occur with development of reading. This paper will examine the role of reading acquisition on the development of visual processing skills in a unique and novel way on a rare longitudinal dataset. The use of explanatory item response models allows us to uniquely study the interplay of task demands, as measured by item features, and student characteristics, as measured by time varying covariates of reading and reading related skills, to understand how the development of reading affects the development of visual processing as measured by standard educational and neuropsychological tests.

### THE EXPLANATORY ITEM RESPONSE MODELS

Application of explanatory item response models to analyze item level data has gained significant interest among psychometricians, statisticians, and educational researchers over the last decade. The models became popular because of their focus on explaining item responses on a test in terms of: (a) the effects of person characteristics on person abilities (θ_p_ – one’s location on a latent trait continuum), as well as (b) the effects of item features on item difficulties (β_i_ – difficulty of an item designed to measure some latent ability; de Boeck and Wilson, 2004). In other words, these models attempt to jointly explain a person’s position on the ability dimension as a function of person characteristics, and an item’s position on the difficulty dimension as a function of item features. Consequently, external variables explain individual differences in responses to test items through their influence on ability and item difficulty. In many applications, one-parameter (1PL) variants of the explanatory item response models are preferable over other item response models [e.g., two-parameter (2PL) or three-parameter models (3PL)]. The 1PL model constrains the relationship between item performance and ability, referred to as item discrimination, to be the same for all test items and allows item difficulty to vary across items. Thus, items differ from one another only in terms of how difficult they are. Placing a constraint on the discriminability parameter carries important implications for interpretation of the unknown parameters and scoring of the test. Specifically, the restriction implies that the test is unidimensional, measuring a single latent ability, and further implies that the number of correct item responses is a sufficient statistic for person ability, that is, there is a one-to-one mapping between the number correct and person ability. The 1PL model also implies that the probability of correctly answering a more difficult item can never exceed the probability of correctly answering an easier item for individuals of any given ability level. The same is not true for the 2PL and 3PL.

Although the models are quite complex, they can be understood as a multivariate extension of multiple (logistic) regression with dichotomous outcomes. The multivariate extension allows us to capture variation across items within a test and time point as well as variation within and between items that occur in conjunction with development (i.e., change over time). In the current project, application of the 1PL explanatory item response models allowed us to model changes in responses to test items as a function of development and, particularly as a function of changes in person characteristics related to learning to read. That is, we used explanatory item response models to explain variability in item difficulties, in terms of item features, person characteristics, and their interactions over the developmental period where children learn to read from the beginning of kindergarten to the end of second grade in the U.S. If learning to read affects children’s processing of visual information, then these effects should be evidenced by interactions between measures of reading and time in the explanation of item difficulties, that is, the influence of the reading measures on item difficulties will change over time.

## MATERIALS AND METHODS

### PARTICIPANTS

The sample of the current study was drawn from a longitudinal study of students’ development of reading and reading precursor skills (Boscardin et al., 2008). The original project focused on developmental patterns of early reading skills and whether models of individual growth could identify children who were at-risk for the development of reading problems. The current study involved 932 students enrolled in regular educational programs at three elementary schools in the same district in a metropolitan area in Texas. Students were excluded from participation due to severe emotional problems, vision difficulties that were uncorrected, hearing loss, neurological disorders, and lack of proficiency in English as measured by the school district. Students were enrolled in the project beginning in Kindergarten, grade 1, or grade 2 and followed through the end of grade 2. Thus, children enrolled in Kindergarten were followed for 3 years whereas students enrolled in grade 2 were followed for 1 year. Each student was assessed on a variety of reading and reading precursors four times per year (October, December, February, and April) for the duration of their time in the study. Thus, students enrolled in kindergarten were tested as many as 12 times over the course of their participation, whereas children enrolled in grade 2 were tested up to four times on the reading precursors and reading measures. In addition, children were also administered a standardized achievement and intellectual assessment in May of each year at the end of Grade 1 and Grade 2. The mean ages of the students were 5.86 years (SD = 0.36) for the kindergartners, 6.92 years (SD = 0.38) for Grade 1, and 7.98 years (SD = 0.42) for Grade 2. **Table 1** provides the ethnicity and SES for the sample. Socioeconomic status (SES) was measured using the Hollingshead (1975) Four Factor Index of Social Status. This index combines information on mothers’ and fathers’ education and occupation status.

**Table 1:** Demographic characteristics of the sample.

		*N*	%
Gender	Male	468	50.21
	Female	464	49.79
Ethnicity	Caucasian	469	50.32
	African American	161	17.27
	Hispanic	152	16.31
	Asian	141	15.13
	Other	9	00.97
SES	Lower	66	07.08
	Working	356	38.20
	Middle–upper	405	43.45
	Not provided	105	11.27

### MEASURES

The measures assessed from October through April signified constructs thought to be important in the development of early reading skills, which was the focus of the original study that guided the design and data collection strategy. The measures used in this study can be categorized into: (a) visual motor and visual discrimination, (b) precursor and reading-related skills, and (c) norm referenced achievement and intelligence measures. Although in the original study these latter measures were included as possible predictors of reading acquisition and reading problems, in the present study they serve as the outcomes of interest.

### VISUAL MOTOR AND VISUAL DISCRIMINATION

#### Visual-motor integration (VMI)

Visual-motor abilities (specifically VMI) were assessed using the Beery Test of Visual Motor Integration (VMI third edition; Beery, 1989). This instrument is a paper and pencil test, which required students to copy 24 geometric line drawings of increasing difficulty without using erasures. All students start with the first item and continue until a ceiling of three consecutive failures is reached. Inter-rater reliability has been reported at 0.93 with a median split-half reliability of 0.79. This measure was administered from kindergarten through Grade 2. The raw scores range from 0 to 24.

#### Recognition–Discrimination (RecDis)

Perceptual discrimination, measured by the Recognition–Discrimination Test (Satz and Fletcher, 1982), is a visual perceptual (matching) task. The students are required to identify a geometric stimulus design differing among a group of four figures, three of which were rotated and only one, the target, was similar in shape to the stimulus figure. The test is timed, and has three practice items and 21 test items. This instrument was included in this study as an additional non-linguistic measure since it is motor free, has good reliability (Kuder–Richardson coefficient of 0.94), and has demonstrated good predictive validity for reading group classification throughout elementary school (Satz et al., 1978). This measure was administered from kindergarten through Grade 2. The raw scores range from 0 to 21.

### PRECURSOR AND READING-RELATED SKILLS

#### Phonological awareness (PA)

Phonological awareness was measured using a prepublication version of the Comprehensive test of phonological processes (CTOPP; Wagner et al., 1999). For this study, students’ PA was estimated based on an item response theory (IRT) model involving six of the seven subtests in the battery. The seven subtests included *blending onset and rime*, *blending phonemes into words*, *blending phonemes into non-words*, *first-sound comparison*, *phoneme elision*, *phoneme segmentation, and sound categorization*. According to Schatschneider et al. (1999) the sound categorization subtest provided little information about PA since it did not discriminate well between students at different ability levels. Therefore, this subtest was excluded from the study when estimating students’ PA scores. Internal consistency estimates for the subtests as reported by Wagner et al. (1993) ranged from 0.71 to 0.87 over the subtests, and estimates calculated in the present study ranged from 0.85 to 0.95. Instead of using raw total scores of phonological ability, scores were expressed as IRT-model-based estimates of each student’s latent phonological ability to represent PA with a mean of 0 and SD of 1. These measures were administered from kindergarten through Grade 2.

#### Rapid serial naming (RAN)

Rapid naming was assessed through administration of Denckla and Rudel’s (1976) Rapid Automatized Naming (RAN) tests for objects and letters. The task requires children to name familiar objects or letters within a set time. The Rapid Naming of Object (RNO) task consisted of line drawings of common objects (i.e., flag, drum, book, moon, and wagon); the Rapid Naming of Letters (RNLs) task consisted of high-frequency lower-case letters (i.e., a, d, o, s, and p). For each task, the stimuli consisted of two practice items and five test items repeated 10 times in random sequences. The child was asked to name each stimulus as quickly as possible. The correct number of responses named within 60 s was recorded. Test–retest reliability was 0.57 for kindergarten (reflecting variability in true change over this age range) and 0.77 for Grades 1 and 2 (Wolf et al., 1986). Test–retest reliability was 0.87 for RNL and 0.76 for RNO when the test and retest were 2 months apart. In this study, RNO and RNL were administered from kindergarten through Grade 2.

#### Word reading (WR)

Students were presented a list of 50 words on 3 × 5 index cards. Words were presented one at a time and the student was asked to read each word as it was presented. This measure was administered four times per year, but only in first and second grade. There were 16 words that were included on both the first and second grade test forms. Thus, across the two forms, a total of 84 words were used, with 16 words in common and 34 words unique to grade one and 34 words unique to grade 2. The 50 words on either form included 36 single-syllable, 11 two-syllable, and 3 three-syllable real words. For the present study, word-reading ability was estimated using a 2PL model for the item responses (Hambleton et al., 1991). Scores were expressed as IRT model- based estimates of each student’s latent ability and were scaled to have a mean of 0 and SD of 1 across grades 1 and 2. Internal consistency estimates calculated in the present study exceeded 0.90 on all occasions.

#### Spelling

Children in Grades 1 and 2 were presented the same list of 50 reading words and asked to write them on a sheet of paper. Of the 50 words, 32% had four letters, 40% had five letters, 18% had six letters, and 10% had seven letters. Half had predictable spelling patterns and half had unpredictable spelling patterns. Words were presented alone and in a sentence. The spelling test was administered in a group format in the students’ regular classrooms. Words were presented in blocks of 10 over a period of 5 days. All other tests were individually administered. Scores were expressed as IRT-model-based estimates of each student’s latent ability and were scaled to have a mean of 0 and SD of 1. Internal consistency estimates calculated in the present study exceeded 0.85 on all occasions for this subtest.

#### Word reading fluency (WRF)

In the pre-publication version of the Test of Word Reading Efficiency (TOWRE: Torgesen et al., 1999), students were presented with a word list containing 104 words divided equally into four columns. Students were directed to read the words as fast as they could and were given a short eight-item practice list first. Two items were recorded during this reading, the total number of words read and the total number of words read correctly within the 45 s time limit. In order to estimate students’ word reading fluency, their total correct score from the word-reading test (WR) was divided by the total time (45 s).

#### Vocabulary (PPVT)

The Peabody Picture Vocabulary Test–Revised (PPVT-R; Dunn and Dunn, 1981) was administered to assess oral vocabulary levels of children from kindergarten through Grade 2. The PPVT-R is a well-established measure for receptive vocabulary. For this measure, the child is presented with a stimulus word and then shown a set of four pictures. The child is then asked to choose the picture that represents the word.

### NORM REFERENCED ACHIEVEMENT AND INTELLIGENCE MEASURES

At the end of Grades 1 and 2, standardized measures of academic achievement and intelligence were administered. For the purposes of this study, the results of the Woodcock–Johnson-Revised subtests and the Hobby short form of the Wechsler Intelligence Scale for Children-Revised (WISC-R) are reported to provide information on the general abilities of the study sample. These measures are not otherwise used in the analyses.

#### Woodcock–Johnson psycho-educational test battery-revised

The Woodcock–Johnson battery includes several tests for measuring skills in reading, mathematics, and writing, as well as important oral language abilities and academic knowledge. However, only three of the subtests were used for the purpose of this study.

#### Woodcock letter word identification (WJR:WI)

This measure assesses the child’s ability to decode isolated words of varying difficulty. In this subtest, students are required to first identify letters, which are presented in large type, and then to pronounce the presented words correctly (Woodcock and Johnson, 1989).

#### Woodcock word attack (WJR:WA)

This subtest measures grapheme-to-phoneme translation of pseudo words that are not contained in the lexicon. In this subtest students are required to provide sounds for single letters and to read combinations of letters that follow English orthographic rules but are either low frequency or non-sense words (Woodcock and Johnson, 1989).

#### Woodcock passage comprehension (WJR:PC)

This subtest consists of three item types and is a general measure of reading comprehension. The first item type has the student match a pictographic representation of the word with an actual picture of the object. The second type provides a multiple-choice format for which the student is asked to point to the picture represented by a phrase. Finally, the student reads a short passage and identifies a missing key word that fits within the context of the passage.

These measures are highly reliable with internal consistency estimates above 0.90 and extensive demonstrations of validity (Woodcock and Johnson, 1989). These subtests are normed to a mean of 100 and a SD of 15 in each grade.

#### Wechsler intelligence scale for children-revised (WISC-R)

Students were administered the Hobby short form (Hobby, 1982) of the WISC-R (Wechsler, 1974). The WISC-R was standardized on a large sample of children, ages 6.0–16.5 years, stratified for age, gender, race, and SES according to 1970 U.S. census information. Test–retest reliabilities for all tasks ranged from 0.73 to 0.95. The average correlations among Stanford-Binet IQ scores and WISC-R Verbal, Performance, and Full-Scale IQs were 0.71, 0.60, and 0.73, respectively.

#### Hobby short form

The Hobby short form (Hobby, 1982) was used because of the large number of children participating in the study. While the form contains all the subtests of the WISC-R, the administration is limited to every other item, with raw scores adjusted for the items that were omitted by design. The correlation between IQ scores from the full WISC-R and the Hobby short form are at 0.98 and above (Hobby, 1982; Sattler, 1993).

#### WISC-R performance IQ (WISCP)

This score reflects non-verbal intelligence as measured by five subtests: Picture Completion, Digit Symbol, Picture Arrangement, Block Design, and Object Assembly.

#### WISC verbal IQ (WISCV)

This test focuses on language-based skills and includes six subtests: information, similarities, arithmetic, vocabulary, comprehension, and digit span.

### DATA ANALYSES

The cross-classified linear logistic test models with separate random intercepts for people and items were used to determine whether variation in item difficulties for test items from the two visual processing measures (VMI and RecDis) could be attributed to developmental growth in reading ability or due to maturation unrelated to reading as reflected simply by students’ age. The models had a cross-classified random effects structure to deal with dependencies among the responses to items as these dependencies result from administering all items to all students with all students responding to all items. That is, item responses were cross-classified in persons and items. Specifically, (a) the first-level of the model included responses to items (dichotomous variables coded 0 or 1, where 1 = correct, 0 = incorrect), (b) the second-level included item and person parameters which are crossed in the design. In all models, person and item parameters were random (as reflected by random intercepts), whereas effects of person and item characteristics were fixed.

A hierarchical modeling approach was used to address the study hypotheses. At the first stage, a descriptive model of item difficulties for test items from the two visual processing tests was developed. After that, explanatory item response models were utilized to explain variation in item difficulties and the effects of item features on item difficulties through moderating effects of person characteristics, and changes in person characteristics over time. These interaction parameters that examine changes in the effects of person characteristics over time capture the effects of interest. Specifically, these interaction parameters test whether learning to read changes how children process visual motor and visual discrimination test items. Maximum likelihood estimation based on Laplace approximation was used to estimate all unknown model parameters. All models were estimated utilizing the *glmer* function of *lme4* package in *R* (Bates et al., 2008) as this function is suitable for estimating models with random effects and cross-classified structure.

## RESULTS

**Table 2** reports descriptive statistics including means and SDs for the achievement and intellectual measures among first and second graders as the standardized achievement and intelligence tests were not administered in kindergarten. **Tables 3 and 4** present descriptive statistics for reading and reading precursor measures with respect to different time points from the beginning of kindergarten through the end of second grade.

**Table 2:** Descriptive statistics for achievement and intellectual measures.

Measure	Grade 1		Grade 2	
	*M*	SD	*M*	SD
Woodcock reading comprehension	105.70	14.84	107.04	14.97
Woodcock letter–word identification	106.83	16.66	107.21	17.09
Woodcock word attack	104.26	15.42	103.53	16.02
WICH performance IQ	111.83	14.60	113.78	14.18
WISC verbal IQ	104.48	14.20	106.66	14.75

*A close analysis of the data shows that students’ mean performance on all measures has increased across the different time points over a 3-year period. This pattern of finding suggests that students’ reading and reading precursor skills develops as a function of age. A close analysis of the data suggests that students’ mean performance, with the exception of WJWA, have increased across the grade levels.*

**Table 3:** Descriptive statistics for kindergarten data collected longitudinally.

Wave	0		1		2		3	
Measure	*M*	SD	*M*	SD	*M*	SD	*M*	SD
VMI	9.3	3.3	10.6	3.8	11.0	3.7	11.9	4.2
Recognition–Discrimination	12.5	3.9	13.8	3.5	14.7	3.2	15.2	3.1
Age in months	67.5	3.7	69.3	3.8	71.3	3.7	73.4	3.7
Phonological awareness	-1.2	0.6	-1.0	0.7	-0.8	0.7	-0.6	0.8
Rapid Naming of Letters	0.5	0.4	0.7	0.4	0.8	0.4	0.8	0.4
Rapid Naming of Objects	0.6	0.2	0.7	0.2	0.7	0.2	0.7	0.2
Vocabulary	55.9	15.0	57.7	14.8	62.0	15.1	64.4	14.6

*VMI, Beery visual motor integration.*

**Table 4:** Descriptive statistics for grade 1 and 2 data collected longitudinally.

Wave	4		5		6		7		8		9		10		11	
Measure	*M*	SD	*M*	SD	*M*	SD	*M*	SD	*M*	SD	*M*	SD	*M*	SD	*M*	SD
VMI	13.6	4.7	14.1	4.9	15.1	5.2	15.4	5.1	18.0	6.3	18.9	6.9	19.3	7.2	19.8	7.2
Recognition–Discrimination	15.8	2.8	16.6	2.8	17.1	2.7	17.5	2.5	17.6	2.4	18.3	2.1	18.4	2.1	18.8	1.9
Age in months	80.2	4.1	82.0	4.1	84.1	4.1	86.1	4.1	92.9	4.6	94.7	4.6	96.8	4.6	98.8	4.6
Phonological awareness	-0.2	0.7	0.1	0.7	0.3	0.7	0.5	0.7	0.5	0.6	0.7	0.6	0.8	0.7	1.0	0.7
Rapid Naming of Letters	1.1	0.4	1.2	0.4	1.3	0.4	1.4	0.4	1.6	0.4	1.7	0.4	1.7	0.4	1.8	0.4
Rapid Naming of Objects	0.8	0.2	0.9	0.2	0.9	0.2	0.9	0.2	1.0	0.2	1.0	0.2	1.0	0.2	1.0	0.2
Vocabulary	72.3	13.9	74.2	14.4	77.9	14.2	79.7	14.3	86.1	13.8	86.8	13.8	89.9	13.6	91.5	13.6
Word reading	-0.9	0.8	-0.6	0.9	-0.3	0.9	-0.1	0.9	0.3	0.7	0.5	0.7	0.7	0.7	0.8	0.7
Reading efficiency*	0.3	0.3	0.4	0.3	0.5	0.4	0.6	0.4	0.8	0.4	0.9	0.3	1.0	0.3	1.0	0.3
Spelling	-0.9	0.7	-0.6	0.8	-0.4	0.8	-0.1	0.8	0.3	0.6	0.6	0.6	0.7	0.7	0.8	0.7

VMI, Beery visual-motor integration; *words per 45 s.

**FIGURE 1:**
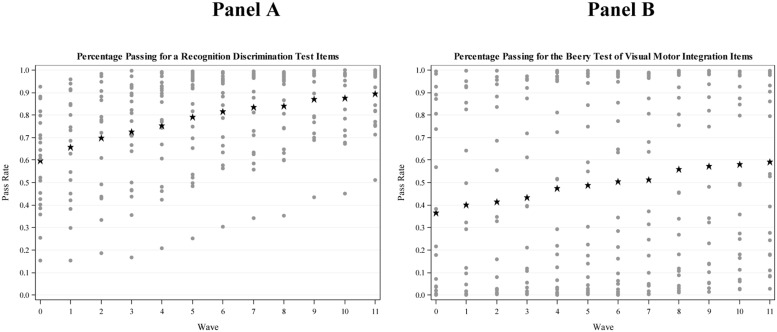
**Dot plots demonstrating the percent correct for the Recognition–Discrimination Test **(A)** and the Beery Test of Visual Motor Integration **(B)** over time.** Dots were used to represent the percent correct for each item at any given point in time. The stars plotted in each panel represent the average percent correct across all items at each wave.

**Figure 1** presents the pass rates (% correct) for the RecDis and VMI items in Panel A and B, respectively, as a function of item features and time from the beginning of kindergarten through the end of grade 2. The pass rates for VMI and RecDis items were estimated based on the frequencies of correct responses for each item at the 12 time points. Each point on the graph depicts the percentage of correct responses for a particular item at a particular wave of data collection. Also depicted on the figure is the average percent correct across all items, shown in each panel as a star at each time point. The panels show that, for both tests, the pass rates were gradually increasing over time indicating the developmental trajectory of visual processing skills. The panels also show that, on average at any given point in time, VMI items were more difficult than RecDis items in that the average percent correct was lower and variation in test scores was greater for VMI items.

These initial findings were further explored using two explanatory item response models to further clarify the features of items that affect item difficulty. In the first model (model 1), wave, item type (VMI vs. RecDis), and the interaction of wave and item type were included as explanatory variables. In the second model (model 2), a more complete classification of item types was included. In particular, we classified items into five categories: (a) motor vs. non-motor, and then further distinguished among four types of motor item, (b) closed geometric designs, (c) closed designs comprised of simple horizontal and vertical lines, (d) open geometric designs with acute and oblique angles, and (e) closed geometric designs having three-dimensional features. This classification of the motor items was based on the theory underlying the development of visual processing skill in children, which undergirds the development of the VMI test. As in Model 1, wave, item type, as well as the interaction of wave and item type were used as explanatory variables.

**FIGURE 2:**
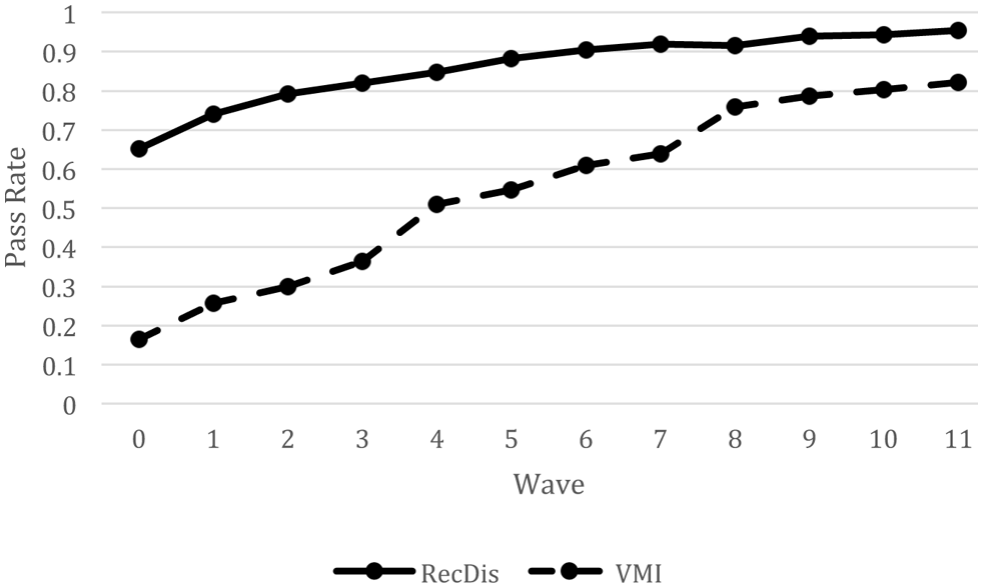
**A line plot demonstrating a pass rate for items with and without significant motor demands over time.** RecDis, items without significant motor demands; VMI, items with significant motor demands.

**Figure 2** presents the results for Model 1 and highlights the differential effects of time for the RecDis and VMI test items. The model was estimated to capture any difference in the developmental time course for motor and non-motor visual processing items. This figure makes clear that differences between motor and non-motor items in the estimated percent correct from models 1 became smaller across the 12 time points. Although items without motor demands were easier at each wave and became easier over time, the difference between motor and non-motor items became smaller at each wave. That is, the average percent correct was increasing more rapidly for motor-based items than for items without significant motor demands, at least in part because of the overall higher performance on non-motor items. This pattern is not uncommon in learning data, namely that the rate of progress slows as the room for progress diminishes.

**FIGURE 3:**
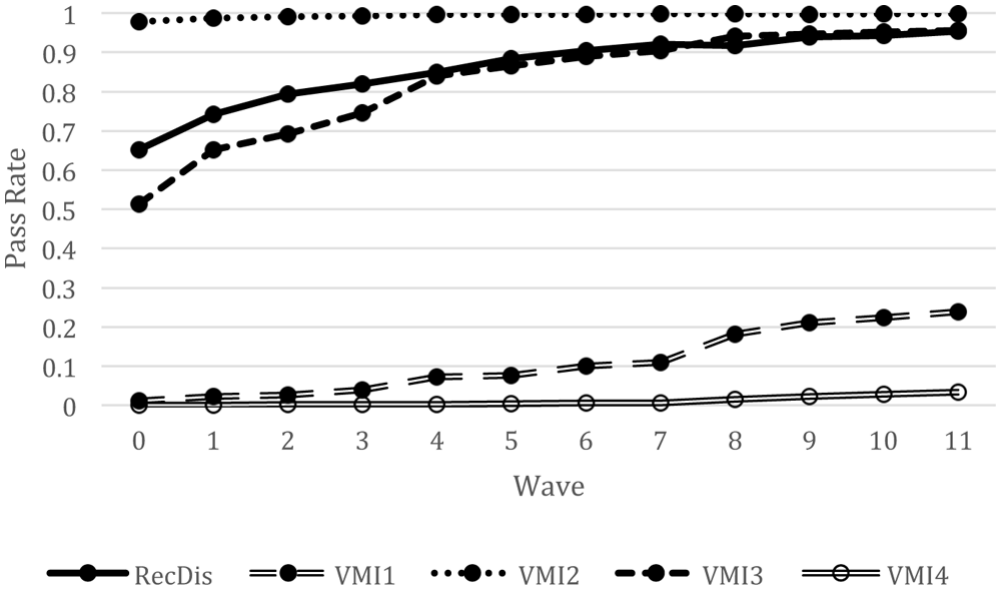
**A line plot demonstrating a pass rate for items with different structural features of design over time.** RecDis, items representing rotated line drawings; VMI1, items representing closed geometric designs with acute and oblique angles; VMI2, items representing closed geometric designs comprised of simple horizontal and vertical lines; VMI3, items representing open geometric designs with acute and oblique angles; VMI4, items representing closed geometric designs having a three-dimensional quality.

**Figure 3** presents interactions between item type and wave for RecDis and VMI tests, with VMI items differentiated according to various item features. As mentioned above, we distinguished between motor and non-motor items and further distinguished among motor items representing closed geometric designs, closed designs comprised of simple horizontal and vertical lines, open geometric designs with acute and oblique angles, and closed geometric designs having three-dimensional features. These four distinguishing characteristics of the VMI items were related to increased item difficulty, as is evidenced clearly in **Figure 3**. Specifically, items representing closed geometric designs with acute and oblique angles, or having three-dimensional quality were the most difficult on average. At the same time, items representing closed geometric designs comprised of simple horizontal and vertical lines had a pass rate of nearly 100% indicating very low difficulty for these items. More importantly, the development of visual processing skills varied according to these structural features as evidenced by differences across time in estimated pass rates for items with different features. In particular, the developmental trajectory of visual processing skills was observed to be essentially flat and near 100% for VMI items consisting of closed figures comprised of vertical and horizontal lines. Similarly, the developmental trajectory for VMI items representing closed geometric figures of a three-dimensional nature was relatively flat, but in this case the percent passing for items of this type was essentially zero. The developmental trajectories for RecDis and VMI items comprised of closed designs with acute and oblique angles were almost identical, with slightly higher pass rates for the RecDis items in kindergarten (waves 0–3) and no difference between the two trajectories from wave 4 through 12. Finally, items on the VMI that represented closed geometric designs with acute and oblique angles showed a somewhat different pattern over the 12 waves. For these items, the pass rate increased steadily from about 5% at the end of kindergarten to between 20 and 30% by the end of grade 2.

These developmental differences in the pass rates across item types are interesting, but they do not, in and of themselves, indicate that item performance is changing because of the onset of learning to read. To test the primary hypotheses about developmental effects of reading acquisition on visual processing of test items, we ran a series of models looking at the effects of person characteristics on ability estimates, and most importantly, examining interactions between person characteristics, item features, and time. We began with estimating classes of models where reading measures were added to models 1 and 2. Specifically, each reading measure was added individually to the model along with IQ, wave, item type, the two-way interaction of wave and item type, as well as the three-way interaction of reading measure, wave, and item type. We ran models using each of the two ways of coding item type: (a) distinguishing motor (VMI) from non-motor (RecDis) items, and (b) differentiating among the five categories of item just described. These models were computed in order to explain variation in item difficulties and the effects of item features on item difficulties as a function of person characteristics, and changes in person characteristics over time.

**Table 5** shows the estimated pass rates at the end of grades 1 and 2 from the set of models just described. These models were estimated using first and second grade data but not kindergarten data because word reading, spelling, and reading fluency were not administered during kindergarten. As can be seen from **Table 5**, these models revealed statistically significant main effects of PA, RNL and objects, vocabulary, word reading, reading efficiency, and spelling over and above other predictors. Most importantly, person characteristics did not interact with wave in a statistically significant way. Students with higher reading and reading related skills performed better on visual processing tests, but these effects did not change with time. In other words, there was a generalized ability related difference in performance on visual processing tests, but this difference did not vary with development, nor did it vary systematically as a function of item type and wave. This pattern of findings is inconsistent with the hypothesis that development of reading changes how students process visual information.

**Table 5:** The influence of individual abilities on the probability of correctly answering an item of average difficulty.

	End of grade 1 (Wave 7)		End of grade 2 (Wave 11)	
Measure	PR-LA	PR-HA	PR-LA	PR-HA
**Recognition–Discrimination Test**
Phonological awareness*	0.916	0.949	0.947	0.967
Rapid Naming of Letters*	0.930	0.940	0.956	0.966
Rapid Naming of Objects*	0.931	0.940	0.961	0.965
Vocabulary*	0.926	0.947	0.960	0.965
Word reading*	0.922	0.946	0.947	0.965
Reading efficiency*	0.931	0.952	0.955	0.969
Spelling*	0.926	0.946	0.948	0.965
**Beery test of visual motor integration**
Phonological awareness*	0.627	0.742	0.732	0.820
Rapid Naming of Letters*	0.674	0.706	0.772	0.812
Rapid Naming of Objects*	0.677	0.708	0.793	0.810
Vocabulary*	0.658	0.735	0.787	0.811
Word reading*	0.652	0.736	0.738	0.815
Reading efficiency*	0.638	0.722	0.736	0.805
Spelling*	0.661	0.732	0.738	0.812

*p < 0.001; PR-LA, pass rates for students with low ability; PR-HA, pass rates for students with high ability; *N* = 762. Pass rate is the estimated probability of a correct response on an item of average difficulty on a particular assessment. Models control for performance IQ, wave, item type, wave-item type interaction, and wave-item type-person characteristic interaction. Each model include only one person level characteristic.

It is important to point out that the models reported in **Table 5** yielded identical findings in terms of estimated passing rates for specific person characteristics regardless of whether item type distinguished only motor items from non-motor items, or distinguished among the different item features depicted in **Figure 3**. This outcome was not surprising as person and item features were included in these models in a manner such that person characteristics explained person ability whereas item features explained item difficulty.

In looking at the effects of individual person characteristics in **Table 5**, it is important to also keep in mind that the models reported in **Table 5** examined the effects of person characteristics individually. Because these characteristics are correlated with one another, the possibility exists that these effects are overlapping and are not unique to the individual predictors listed in the table. To determine which person characteristics exert the largest independent influence on visual processing abilities, we next examined models that incorporated multiple person characteristics simultaneously. These models showed that several of the effects reported in **Table 5** are redundant. Specifically, we found that PA and spelling seemed to exert independent effects over and above the other predictors. That is, once PA (*b* = 0.21, *SE* = 0.02, *p* < 0.001), word reading (*b* = 0.08, SE = 0.04, *p* < 0.05) and reading efficiency were included in the same model, reading efficiency was no longer statistically significant (*b* = 0.07, SE = 0.04, *p* = 0.07). Additionally, the effect of word reading was negligible when spelling (*b* = 0.08, SE = 0.03, *p* < 0.05) was included along with PA (*b* = 0.20, SE = 0.02, *p* < 0.001), word reading (*b* = 0.05, SE = 0.04, *p* = 0.13) and reading efficiency (*b* = 0.04, SE = 0.04, *p* = 0.33). As such, PA and spelling were the most important, unique, predictors of performance on visual processing tests.

Most importantly, although these person characteristics related to visual processing abilities, there was no consistent evidence to suggest that abilities related to reading interacted with item type and wave in their effects on visual processing. Although individual interaction terms were occasionally statistically significant at a nominal alpha level of 0.05, they did not meet the adjusted alpha level set by the False Discovery Rate (FDR) of Benjamini and Hochberg (1995). Note, the FDR is generally regarded to be the most powerful approach to multiple comparisons when many hypotheses are being tested, and is thus preferred in this context over other multiple comparison procedures. It is also noteworthy that the significant interaction effects typically involved a single wave and item feature, and did not reflect a developmental pattern. For these reasons, we conclude that those interactions with significant nominal *p*-values and non-significant adjusted *p-*values constituted false rejections/false discoveries and should not be viewed as statistically significant. In that sense, we found no evidence to suggest that reader characteristics interacted with item-type and wave to differentially affect item difficulties as children acquired reading skills. In short, the findings support the idea that visual processing skills are related to the person abilities listed in **Table 5** and uniquely related to PA and spelling, but they are not consistent with the idea that learning to read changes how children process visual information as we found no consistent evidence for differential effects of person characteristics over time.

## DISCUSSION

Interest in the role of visual processing in reading is not new and is not surprising. Reading is, at first glance, a visual task when performed by individuals with normal or corrected vision. However, the role of visual processing skills in reading have been found to be relatively minor, in so far as differences in visual processing skills do not explain variation in reading performance once skills related to the linguistic basis for reading have been taken into account. That is not to say that visual skills are unimportant in reading, but that individual differences in visual processing do not account for individual differences in reading performance. In learning to read, children must learn the process for transforming graphical inputs into spoken language. While the visual features of writing systems present some challenges to beginning readers, they pale in comparison to the challenge of abstracting the sound features of a spoken language from the writing system. Indeed, the importance of visual skills in reading has been shown experimentally through eye movement research and studies that control the flow of visual information to the reader (Rayner, 1998). It is without question that vision plays a crucial role in the cognitive processes involved in reading. However, it seems also to be the case that individual differences in visual processing explain little of the heterogeneity in reading acquisition (Fletcher et al., 1999). The present study contributes to research in the areas of visual processing and reading by taking a unique look at how reading contributes to the development of visual processing. The study made use of recent advances in the statistical modeling of item responses through cross-classified random effects models for binary outcomes. Specifically, we applied these models, known as explanatory item response models, in a developmental context during the early acquisition of reading skill to examine the characteristics of individuals that explain visual processing ability, the characteristics of test items that explain item difficulty, and most importantly, to investigate the presence of cross-level interactions between reader characteristics and item features which would signal that learning to read was altering the ways in which students relate to test items measuring visual processing ability. Despite finding significant and substantial effects of various item design features on item difficulty, as well as finding various subject characteristics that related to persons’ ability to perform on test items, we found no consistent evidence for the presence of interactions which would have signaled that learning to read was differentially affecting the difficulty of tests items over time.

Rather than suggesting that learning to read altered the measurement of visual processing, results simply suggested that individuals’ characteristics as readers explained some of the variability in visual processing abilities, but these relationships were not moderated by development or by item features. Study results were consistent with other research on the developmental sequence of visual motor (VMI) and motor-free (RecDis) visual processing skills in that item difficulty varied according to the type of figure presented. It was also the case that motor-free items (RecDic) were generally easier for students than visual motor (VMI) items. These results corroborate earlier research on the development of visual processes in children, and earlier factor analytic work on the VMI which showed that tests of visual motor performance are not, necessarily, one-dimensional (Polubinski et al., 1986; Brown et al., 2009).

Given that the research literature is sparse in either describing or explaining how phonological abilities and/or reading *per se* affect the processing of visual information, the results of the present study cannot be viewed as definitive. For one, a major limitation of the present study was the focus on operational tests of visual processes, rather than using carefully controlled or precise measures of visual processing that might tie more closely to the neural basis for visual processing skills. It is quite possible that measures of brain cortical activity, or precise measures of speed of processing of visual information might have revealed more subtle effects of learning to read on the processing of visual information, in much the way that research with neuroimaging techniques has found evidence of changes to visual processing areas following the onset of reading. At the same time, the current study employed a large sample and extensive longitudinal follow-up, so it is difficult to attribute the lack of findings to low power, imprecision in estimating item parameters, or limited change in individuals’ reading and/or visual processing abilities. Both visual processing and reading/reading-related abilities changed substantially over the 3 years from the start of kindergarten through the end of grade 2. Indeed, students went from being non-readers at the start of kindergarten to being proficient beginning readers over this period, with marked variability across children. Similarly, **Figures 1–3** show that there was marked variability in item difficulty across this developmental period, and that much of this variability related to characteristics of the items.

That variation in item difficulty across waves was not related to variation in person abilities in reading and/or reading precursors over this period suggests that the relationships that have been reported in the literature may reflect a failure to adequately control other common sources of variability, such as maturation or increased efficiency/automaticity in reading and related skills. Work by Dehaene et al. (2010) found that the automatic processing of faces in visual association cortex is subject to competition following the acquisition of reading. However, their electrophysiological findings were not corroborated in that study by behavioral findings suggesting that cognitive performance was negatively impacted commensurate with the eletrophysiological evidence of competition.

Together results from neuroimaging studies are not incompatible with those from the present study and its placement within the broader literature on the potential effects of learning to read on visual processing. Rather, the current findings simply serve to highlight that subtle differences in measures of brain electrophysiology are not always consequential for cognition as measured at more macro levels of organization and execution. The history of neuropsychological assessment is rife with examples of behavioral measures failing to differentiate among individuals with gross brain anomalies. Although prior to the advent of non-invasive imaging, neuropsychological and behavioral assessment were the primary means of differentiating organic from functional disease origins, the challenge of showing behavioral correlates of brain electrophysiology remains substantial and prone to statistical artifacts (Vul et al., 2009). At one level, the problems identified by Vul et al. (2009) reflect a problem of sampling bias that inflates estimated relationships. At another level, the challenge of identifying such brain–behavior relationships is one of scale and the fact that true effect sizes in the behavioral and health sciences are often small, making replication an important, but too often neglected component of research (Ioannidis, 2013).

We fully expected that measures of rapid naming, decoding fluency, and spelling, would be most influential in explaining differences in item difficulties and, more importantly, in explaining changes in item difficulties over time. However, we found no such evidence for either prediction. We expected that, as students became proficient in distinguishing strings of graphemes, or words, with increased fluency, students would also become more proficient in discriminating more complex shapes from one another, and in analyzing and reproducing more complex visual stimuli. Contrary to expectations, higher student reading performance simply meant better performance on visual processing skills, and these effects were consistent over time.

Quite clearly, the study design was capable of detecting effects of person abilities on item parameters. We were able to show differences in item parameters over time as small as 0.09 on the logit scale, a difference of about 2.2% in the percentage of correct responses. Clearly, relatively small effects were discernible in the models given our relatively large sample of over 900 students and the extensive longitudinal follow-up of up to 12 observations per individual. That is not to say that all such differences that were small in size could be detected in the models, as effects in the models were correlated. However, it is clear that, for many item types, there was substantial power for detecting meaningful influences of learning to read on item difficulty over time. The failure to obtain such results consistently implies, at a minimum, that such effects on measures of this type must be small, indeed, if they exist at all.

Models involving PA and spelling as predictors found some evidence that these measures exerted unique effects on visual processing abilities. Although numerous predictors explained some of the variability in person ability, most of these effects were redundant with one another, with the exception of spelling and PA. Spelling may have been predictive of visual motor processing as spelling incorporated motor skills at a level of complexity that paralleled that found in the VMI. For instance, when writing words to dictation, stimuli differ substantially in the writing demands they impose on students. For example, writing the letter ‘l’ is easier than writing the letter ‘m’ which is easier than writing the letter ‘q.’ Importantly, both PA and spelling relate to the internal structure of words, which one might expect to relate more closely to visual processing of features. These two contributors to word recognition are known to contribute to the quality of lexical representations, which fuel efficient decoding processes as articulated in Perfetti’s (2007) Lexical Quality Hypothesis (LQH). However, it must also be recognized that, although these measures related to visual processing abilities, they showed no evidence of interacting with item type or wave in affecting item difficulties. This latter point suggests that reading and visual processing abilities are both developing in related ways, but reading abilities do not appear to affect the way that students perform on measures of visual processing. That is to say, children who performed well on measures of reading and reading related skills also performed well on measures of visual processing, and these relations appear to be consistent across the developmental span from the beginning of kindergarten through the end of grade 2 with no indication that reading ability was changing the way in which children performed on the measures of visual processing.

This study set out to review the connection between reading skills as measured by instruments commonly used in academic settings to assess the development of visual motor skill. We did not find evidence that learning to read impacts how children approach these tests. However, it remains possible that findings might differ if alternate measures of visual processing had been used. Measures of sensitivity to information presented extra-foveally or measures of field sensitivity might be expected to show greater influence from learning to read. It is well known that readers process information visually that is outside the area of fixation while reading (Haber and Haber, 1981; Rayner, 1998). Thus, it might be expected that sensitivity to information presented outside the region of primary visual focus would change as children acquire reading. One might predict that while engaged in a reading task sensitivity to the visual features of linguistic information presented extra-foveally would improve as children acquire reading, whereas the same sensitivity might be absent when presented in a non-reading task. This difference would be expected to be smaller for non-readers, and no difference would be expected between readers and non-readers engaged in a non-reading task. Whether effects on visual processing could be obtained on standard paper and pencil tests of visual processing awaits further research, but it seems reasonable to speculate that effects would be more likely to emerge if the visual task more closely approximated reading than either of the current tasks. For example, a task that required individuals to process visually presented information quickly and serially from left to right, or right to left for readers of Arabic and Hebrew, might be more sensitive to learning to read. If such a task could be devised to record responses on a trial by trial basis, then application of the explanatory item response framework could again be used to examine the effects of reader and item features on item performance, and changes in item performance that occur with learning to read (see McBride-Chang et al., 2011). The implications that any such effects might have for teachers and students in school are unclear. However, absent negative effects of learning to read on the processing of visual information in standard educational assessments and tasks, any concern among students, parents, and teachers seems unwarranted.

## Conflict of Interest Statement

The authors declare that the research was conducted in the absence of any commercial or financial relationships that could be construed as a potential conflict of interest.
